# Cybersecurity and Privacy Issues in Extended Reality Health Care Applications: Scoping Review

**DOI:** 10.2196/59409

**Published:** 2024-10-17

**Authors:** Kaitlyn Lake, Andrea Mc Kittrick, Mathilde Desselle, Antonio Padilha Lanari Bo, R Achintha M Abayasiri, Jennifer Fleming, Nilufar Baghaei, Dan Dongseong Kim

**Affiliations:** 1 School of Electrical Engineering and Computer Science, Faculty of Engineering, Architecture and Information Technology, The University of Queensland, General Purpose South, Staff House Rd, St Lucia, 4067, Australia, 61 450 150 234; 2 Department of Occupational Therapy, Royal Brisbane and Women’s Hospital, Metro North Hospital and Health Service, Herston, Australia; 3 Herston Biofabrication Institute, Metro North Health, Brisbane, Australia; 4 Centre for Health Services Research, School of Medicine, The University of Queensland, Brisbane, Australia; ^5^Department of Mechanical, Electrical, and Chemical Engineering, Faculty of Technology, Art, and Design, Oslo Metropolitan University, Oslo, Norway; 6 School of Health and Rehabilitation Sciences, Faculty of Health and Behavioural Sciences, The University of Queensland, St Lucia, Australia

**Keywords:** cyberattacks, cyber defence, cyber security, extended reality, health care, privacy, risk mitigation, virtual reality, cybersecurity

## Abstract

**Background:**

Virtual reality (VR) is a type of extended reality (XR) technology that is seeing increasing adoption in health care. There is robust evidence articulating how consumer-grade VR presents significant cybersecurity and privacy risks due to the often ubiquitous and wide range of data collection and user monitoring, as well as the unique user impact of attacks due to the immersive nature of the technology. However, little is known about how these risks translate in the use of VR systems in health care settings.

**Objective:**

The objective of this scoping review is to identify potential cybersecurity risks associated with clinical XR systems, with a focus on VR, and potential mitigations for them.

**Methods:**

The scoping review followed the PRISMA-ScR (Preferred Reporting Items for Systematic reviews and Meta-Analyses extension for Scoping Reviews), and publications were reviewed using Covidence software. The Google Scholar database was searched using the predefined search terms. The inclusion criteria of the articles were restricted to relevant primary studies published from 2017 to 2024. Furthermore, reviews, abstracts, viewpoints, opinion pieces, and low-quality studies were excluded. Additionally, data on publication statistics, topic, technology, cyber threats, and risk mitigation were extracted. These data were synthesized and analyzed using the STRIDE (spoofing, tampering, repudiation, information disclosure, denial of service, and elevation of privilege) framework, enterprise risk management framework, and National Institute of Standards and Technology Cybersecurity Framework, as well as developing threat taxonomies.

**Results:**

Google Scholar returned 482 articles that matched the search criteria. After title and abstract screening, 53 studies were extracted for a full-text review, of which 29 were included for analysis. Of these, the majority were published in the last 4 years and had a focus on VR. The greatest cyber threat identified to XR components was information disclosure followed by tampering when mapped against the STRIDE framework. The majority of risk mitigation strategies provide confidentiality and integrity and can potentially address these threats. Only 3 of 29 papers mention XR in the context of health care and none of the identified threats or mitigations have been studied in a clinical setting.

**Conclusions:**

This scoping review identified privacy threats where personal and health-related data may be inferred from VR usage data, potentially breaching confidentiality, as the most significant threat posited for health care VR systems. Additionally, immersive manipulation threats were highlighted, which could potentially risk user safety when launched from a compromised VR system. Many potential mitigations were identified for these threats, but these mitigations must first be assessed for their effectiveness and suitability for health care services. Furthermore, health care services should consider the usage and governance of XR for each individual application based on risk threshold and perceived benefits. Finally, it is also important to note that this scoping review was limited by the quality and scope of the studies returned by Google Scholar.

## Introduction

### Background

Extended reality (XR) is a broad term used to refer to augmented reality (AR), virtual reality (VR), and mixed reality (MR), or a technology that combines the use of them. These technologies are defined by their ability to extend the physical world with a virtual world to varying degrees depending on the specific type of XR technology [1]. AR enhances the physical world by overlaying virtual features and functionality, whereas VR immerses the user in a distinct virtual world, often through a head-mounted display (HMD) [2]. Moreover, audio output is also usually present, and together these sensory features immerse the user in a virtual world through visual and audio cues. MR is midway between VR and AR, in which the overlaid virtual world coexists and interacts with the physical one [3]. Each technology provides a new paradigm of human-computer interaction and is designed to improve quality of life, either through entertainment or application in industry and the workplace. A growing area of VR applications is health care [4].

One example of VR applications within health care is in the area of occupational therapy, which aims to improve patients’ quality of independent living through activities and exercises targeting specific movements and functions [5]. Current methods in occupational therapy use different technological resources, such as mechanical setups that emulate real-life activities [6]. Technology-based tools such as video games and sensor-based technologies are also used in clinical practice [7]. Among the XR-based therapy methods, VR systems have shown an increasing demand and have started to be used as an adjunct to conventional therapy [8-10].

However, XR-based health care interventions have cybersecurity and privacy risks. These types of interventions often rely on multiple sensors as input modalities. Many of these sensors (such as eye tracking, microphones, cameras, hand tracking, and motion tracking) are inherit to HMDs and have multiple software components. These components generate an enormous amount of data on a user, which is required to create an immersive experience. However, this monitoring also creates the potential for significant privacy violations if these data are mishandled. When combined with data processing and artificial intelligence (AI) and machine learning (ML) models, this data can be used to identify, deanonymize, and profile users. It has been demonstrated that 100 seconds of VR motion is sufficient to identify a user within a pool of over 50,000 individuals with 94.33% accuracy [11]. A user’s height, wingspan, age, gender, country of origin, and mental and physical disability status are just some additional characteristics that can be inferred from this data [12].

On top of these privacy issues, XR and VR devices are vulnerable to conventional passive and active cybersecurity attacks and threats, including eavesdropping attacks, man-in-the-middle attacks, and denial-of-service (DoS) attacks [13]. These threats can disclose private data or render the XR device unusable. Another type of cyberattack specific to VR includes attacks that target the unique features of an immersive session. They are called immersive attacks and have the potential to cause physical and psychological discomfort or even harm by tampering with the output of the device. For example, they may trick a user into colliding with a real-world object in a chaperone attack by manipulating the display [13], potentially causing the user to fall and injure themselves, or they may display triggering content in an overlay attack [13]. Additionally, cybersickness is a visually induced side effect that can be triggered by these attacks [14]. Thus, the attacks described have been demonstrated in a general experimental setup [13] to have the potential of impacting user well-being by compromising the VR devices.

As XR technology develops, the extent of user tracking and data collection will likely expand and place user security and privacy at further risk (particularly their biometric data). There are 5 categories of countermeasures for these risks that can be found in the literature [3]. They are input protection, data protection, output protection, user interaction protection, and device protection. Despite these countermeasures, no comprehensive mitigating tool exists to protect users from cyberattacks [12].

Although a large and growing body of research exists on the topic of security and privacy issues for XR systems, the specific cybersecurity challenges, risks, and mitigations for XR or VR health care services are underexplored. Most related work is on the risks of integrating Meta [15] XR technologies and ecosystems with health care systems and infrastructure. Meta, formerly known as Facebook, is a company that produces the Meta Quest line of HMDs. They are also responsible for the Metaverse, an XR platform capable of integrating with a range of digital devices and providing multiple use cases and applications, the primary one being social [16]. The key health care services provided by the Metaverse are ubiquitous health monitoring, distributed medical AI, and virtual therapy through gamification and social activities. These services will likely have issues maintaining the privacy and security of user data at rest and in transit over the internet. There is the potential of private and identifying individual data being exposed by AI and ML trained on this data. In addition, gamification exposes a new vector where individuals can be invasively monitored, both by other users in social situations and malicious actors [17]. When the data being gathered and observed is generated from usage in health care services, this can potentially disclose health and biometric information about the user [17,18]. Secure computation techniques such as blockchain are recommended to maintain the integrity and confidentiality of data, along with legal protection of Metaverse-collected data through legislation such as the Health Insurance Portability and Accountability Act in the United States [19]. Although the Metaverse is a growing XR platform, XR technologies are not limited to it.

Apart from Meta, concerns have been raised about the challenges to user privacy rights that come with VR usage in health care. Users can potentially be identified by the extensive data collected by HMD sensors, which might be shared with social media platforms and other parties, like the manufacturers of the device (eg, Meta). This creates technical and ethical challenges for clinical adoption [20]. There is potential danger in using wearable sensors and HMDs due to the amount and type of data generated by them in a clinical scenario, so some health care organizations may not be prepared for the ethical and legal implications [21]. Lakshminarayan et al [22] identified AR and VR technologies as integral to an intelligent, equitable health care system, but these are technologies that can pose significant security and privacy challenges. It is recommended that these be combined with edge computing, wherein computing resources are placed closer to the network to limit transit over the internet.

The existence of cybersecurity and privacy risks of using VR technology in a clinical context has been acknowledged in the literature. However, the specific risks and feasible strategies to mitigate them have not been clearly established. Hence, the potential challenges and consequences of using XR and VR technologies in health care must be understood before widespread clinical adoption to minimize risks to users.

### Objectives

A scoping review was conducted to answer the following two research questions:

- Research question 1 (RQ1): What cybersecurity and privacy risks are there to XR components, in particular VR?
- Research question 2 (RQ2): How can cybersecurity risks to clinical XR systems, in particular VR systems, be reduced?

To determine the potential cybersecurity risks and mitigations for a health care VR system, the cyber threat landscape of XR was first established through RQ1. There are overlaps between different XR technologies in terms of cybersecurity and privacy issues and mitigations, so the investigation was not limited to only VR. Risks applicable to VR components were analyzed and summarized to answer RQ1 by developing taxonomies and using established frameworks to classify and organize XR threats.

To use a VR system in a health care setting, the potential cybersecurity risks and requirements must be understood. RQ2 systematically identifies and analyzes risk mitigation strategies and technologies for the risks established in RQ1, with a focus on risks that uniquely impact XR and VR systems in a health care setting. The results of this scoping review are intended to be used to inform risk management for health care organizations looking to implement XR technology and potential future directions for the secure development of VR tools for use in health care.

## Methods

### Overview

This review was conducted using the PRISMA-ScR (Preferred Reporting Items for Systematic Reviews and Meta-Analyses Extension for Scoping Reviews) checklist (Checklist 1) [23]. Considering the large amount of VR technologies and platforms, VR studies that were not relevant to the HMD platform were excluded. Most literature on the topic of XR cybersecurity and privacy was published after 2017 [24,25]. Additionally, two of the most popular VR devices today, the Meta (formerly Oculus) and HTC Vive line of HMDs, were not released for the public until 2016 [26]. As such, 2017 was chosen as the cutoff year, and any studies published before then were excluded to keep results relevant. Reviews, abstracts, viewpoints, and opinion pieces were excluded, as well as studies where the cyber threats or countermeasures were not relevant to XR or were poorly described. Low-quality studies, including those that have not been peer reviewed, were also excluded. Due to the large number of studies returned by the search terms, the objective was to reduce this to a smaller number of high-quality, relevant primary studies. The full search strategy development and process can be found in Multimedia Appendix 1.

### Database

All literature was found using Google Scholar. Google Scholar is a search engine that indexes content from a range of scholarly sources and databases including the Association for Computing Machinery and Institute of Electrical and Electronics Engineers, as well as medical literature repositories such as PubMed. Due to the research topic’s intersection between different disciplines including computer and medical science, Google Scholar is an effective electronic database to view literature results. This mitigates the risk of missing important literature. Results were refined using the search terms and date range criteria.

### Search Strategy

Google Scholar was searched (one author, KL) from January 1, 2017, to January 1, 2024, using the predefined terms identified in Table 1.

**Table 1.** Google Scholar search term strategy.

Search query	Justification
(privacy OR security OR attack OR threat OR secure OR securing) AND (Virtual reality OR augmented reality OR mixed reality OR extended reality)	Query for privacy and cybersecurity issues in extended reality and related technologies independent of their application
(access control OR side-channel OR user profiling OR tracking user location OR dark designs) AND (Virtual reality OR augmented reality OR mixed reality OR extended reality)	Query for concerns adjacent to privacy and cybersecurity issues independent of their application
(survey architectures OR analysis healthcare) AND (Virtual reality OR augmented reality OR mixed reality OR extended reality)	Query for the use of extended reality and related technologies in health care and nonstandard architectures

The publications returned by this search were screened by title and abstract and for removal of duplicates independently by one author (KL). A full-text review was facilitated by the Covidence research review management software (Veritas Health Innovation [27]) and conducted independently by 3 authors (KL, MD, and AMK) against the inclusion and exclusion criteria. Conflicts were resolved by all authors through discussion and then agreement.

All of the following criteria must be met for a study to be included: (1) publication year is between 2017 and 2024, (2) the issue or mitigation described in the study is relevant and applicable to XR and is well described, and (3) the terms of the search request appear in the title of the study as specified in the full search strategy.

The criteria for a study to be excluded are as follows. Only one of the following criteria must be met for a study to be excluded: (1) the publication date is before 2017, (2) the study is a literature survey, viewpoint piece, or opinion piece, (3) the issue or mitigation described in the study is not relevant or applicable to XR or is poorly described, (4) the study is not peer reviewed, and (5) the study is on VR but is not relevant to the HMD platform.

### Data Synthesis

The complete description of extracted information and the data charting process can be found in Multimedia Appendix 1. Data charting was conducted by one author (KL) into tables using Google Sheets. Disagreements were resolved by discussion, and consensus was reached. The following data were extracted from each included publication: (1) publication information (author and year of publication); (2) topical information (XR domain and topic classification); (3) cyber threat information (cyberattacks identified, attack privilege level and components investigated); and (4) risk mitigation information (defense technologies and risk mitigation strategies identified).

Authors were not contacted to obtain missing data. The complete data extraction template can be found in Multimedia Appendix 1. We used 4 strategies to synthesize and interpret this data: (1) STRIDE threat modelling framework [28] for cyber threat and mitigation analysis, (2) taxonomic summarization of security and privacy issues, (3) enterprise risk management (ERM) [29] mapping of health care concerns, and (4) the National Institute of Standards and Technology (NIST) Cybersecurity Framework [30] for classification of mitigation strategies and technologies.

Cyber threats were categorized using the STRIDE model, which is a common cyber threat modeling framework developed by Microsoft to enable the organization and analysis of cyber threats. STRIDE defines 6 categories of cyber threats: spoofing, tampering, repudiation, information disclosure, DoS, and elevation of privilege [28]. Each threat is explained in Table 2. The threats and cyberattacks extracted from the included publications, if not already categorized by STRIDE, can be given a categorization according to these definitions. Furthermore, the STRIDE framework was used to map mitigating technologies to threats. Each STRIDE threat type is mitigated by the attribute stated in Table 2, and thus mitigations can be classified by the mitigating function they provide.

**Table 2.** STRIDE threat definitions and associated mitigation [28].

STRIDE threat	Definition	Mitigation
Spoofing	Impersonation of other processes, entities, or people can lead to illegitimate access to a system	Authenticity
Tampering	The system can be modified or broken to an adversary’s benefit	Integrity
Repudiation	Actions taken by an adversary in or against the system cannot be traced back to the adversary	Nonrepudiability
Information disclosure	Protected or private information can be disclosed by or to the adversary	Confidentiality
Denial of service	Authentic and permitted users are unable to use the system	Availability
Elevation of privilege	An adversary can illegitimately increase their access to the system	Authorization

Threat taxonomies were developed by manually reviewing each XR-specific cyberattack and organizing them hierarchically based on the following attributes:

1. Security versus privacy: security threats are those that involve an explicit intrusion, modification, or breaking of the system for illicit purposes, while a privacy threat involves data collection with minimal to no illicit intrusion into the system, usually for the purposes of data disclosure.
2. Unique XR feature exploited: the specific XR feature exploited by each threat was identified. These features could include but were not limited to immersive design, communication channels, side channels, and data collection capabilities.
3. Threat target (optional): these threats could be further delineated based on their intended effect, such as manipulation of the user, or intended target, such as the privilege level that the attack targets.

The NIST Cybersecurity Framework is comprised of 5 core functions for organizations to address their cybersecurity measures: identify, protect, detect, respond, and recover [30]. Each function is summarized in Table 3. This framework enables the authors to communicate how and where a mitigation or countermeasure can potentially improve the XR cybersecurity posture of a health care organization. Each mitigation extracted from the included publication can be mapped to a function given the definition.

**Table 3.** National Institute of Standards and Technology cybersecurity framework functions and definitions [30].

Framework function	Definition
Identify	Includes cataloging and understanding the assets, business environment, and risks to the organization, as well as implementing policies and procedures for maintaining cybersecurity
Protect	Regards implementing technical and strategic mitigation strategies to prevent and lower the likelihood and impact of a cyber incident, which may involve implementing access control, awareness and training, and maintaining infrastructure
Detect	Involves implementing technologies and strategies to detect when a cyber incident occurs, which may include implementing continuous monitoring software on assets and detection procedures
Respond	Involves implementing technologies and strategies to respond to a cyber incident, which may include implementing incident response plans and cyber forensic capabilities
Recover	Regards implementing and strengthening recovery ability during or after a cyber incident has occurred, which may include incorporating lessons learned from the incident into policies and procedures and implementing recovery procedures

While the NIST Cybersecurity Framework is effective for communicating mitigations, an ERM framework is effective for articulating and organizing risks to an organization, in this case a health care organization. To this end, 5 categories were selected to articulate organizational risks presented by cyber threats to a health care organization looking at implementing clinical XR systems: compliance with applicable regulatory measures; ability and capacity to deliver health services; confidence of the community and stakeholders in the health care organization; financial performance; and workplace health and safety. Potentially significant risks posed by the threats and cyberattacks identified in the data extraction can be summarized for each category.

## Results

### Search Results

**Figure 1.**
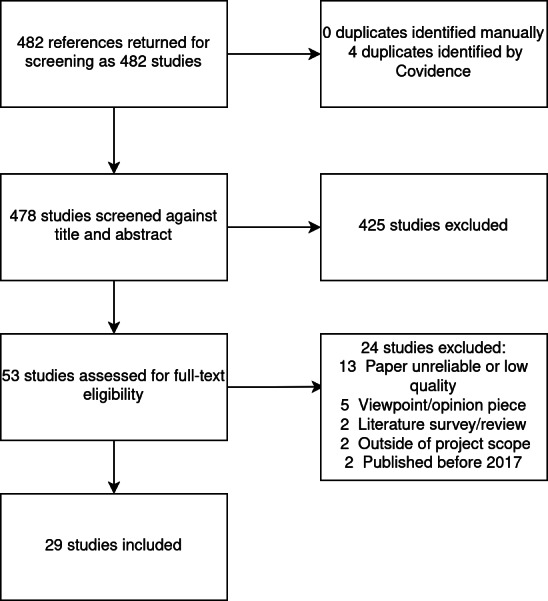
Study extraction and selection process following PRISMA. PRISMA: Preferred Reporting Items for Systematic reviews and Meta-Analyses.

The results of each stage of the search process are summarized in Figure 1. Table 4 summarizes the total number of papers collected and excluded.

**Table 4.** Study collection and exclusion process summary.

	Number of studies
**Search terms used to collect studies**	
(privacy OR security OR attack OR threat OR secure OR securing) AND (Virtual reality OR augmented reality OR mixed reality OR extended reality)	451
(access control OR side-channel OR user profiling OR tracking user location OR dark designs) AND (Virtual reality OR augmented reality OR mixed reality OR extended reality)	13
(survey architectures OR analysis healthcare) AND (Virtual reality OR augmented reality OR mixed reality OR extended reality)	18
**Reasons for study exclusion**	
Abstract screening	425
Paper unreliable or low quality	13
Viewpoint/opinion piece	5
Literature survey/review	2
Outside of project scope	2
Published before 2017	2

### Study Characteristics

#### Publication Statistics

The literature search was restricted to studies published between 2017 and 2024. A total of 482 articles were initially retrieved from the database, and 29 studies were included in the final analysis [31-59] (Multimedia Appendix 2). The publication year distribution can be seen in Multimedia Appendix 3. Most studies found were published in 2023 (10 papers), followed by 2020 (5 papers). Approximately 83% (24/29) of the papers were published in the past 4 years.

#### XR Technologies

The studies were organized according to the XR technology in their title, which could be either VR or any other XR technology (such as AR or MR). A total of 16 studies focused on VR, while the remaining 18 were concerned with other XR technologies. All papers specified the dimension in the title except two works: [59] and [32]. Wang et al [59] was classified as other XR since the study focused on the Metaverse and the paper by Letafati and Otoum [32] was classified as VR. A paper could focus on more than one dimension and so could be counted more than once. A significant number of papers focused on VR (16 papers), but most focused on other XR technologies (18 papers).

#### Cybersecurity Focus

The studies were classified by the contribution they made to the field of cybersecurity and privacy in XR. These contributions were classified as mitigation identified, privacy threat identified, security threat identified, taxonomic analysis, or user experience evaluation. A paper could make more than one contribution and thus have more than one classification. The distribution of contributions can be seen in Table 5. Most papers either identified a mitigation or a privacy threat or both, followed closely by papers that identified a security threat. Taxonomic analyses and user experience investigations were limited to only 5 contributions combined.

**Table 5.** Count of papers organized by contribution.

Contribution	Number of papers
Mitigation identified	18
Novel privacy threat	18
Novel security threat	13
Taxonomic analysis	3
User experience evaluation	2

### RQ 1: What Cybersecurity and Privacy Risks Are There to XR Components, In Particular VR?

The 29 papers covered every XR domain, with a predominant focus on VR. To identify the cybersecurity and privacy issues of XR systems, data were extracted from these papers, such as XR components investigated as well as cyber threats and attacks identified. Taxonomies were developed from the survey data extraction to classify and analyze these data. There are many security and privacy risks to XR components, but the scope was limited to only focus on those that are unique to an XR environment in either their method or effect. Definitions for important cybersecurity and computer science terms used in this section and elsewhere are provided in Multimedia Appendix 4.

**Figure 2.**
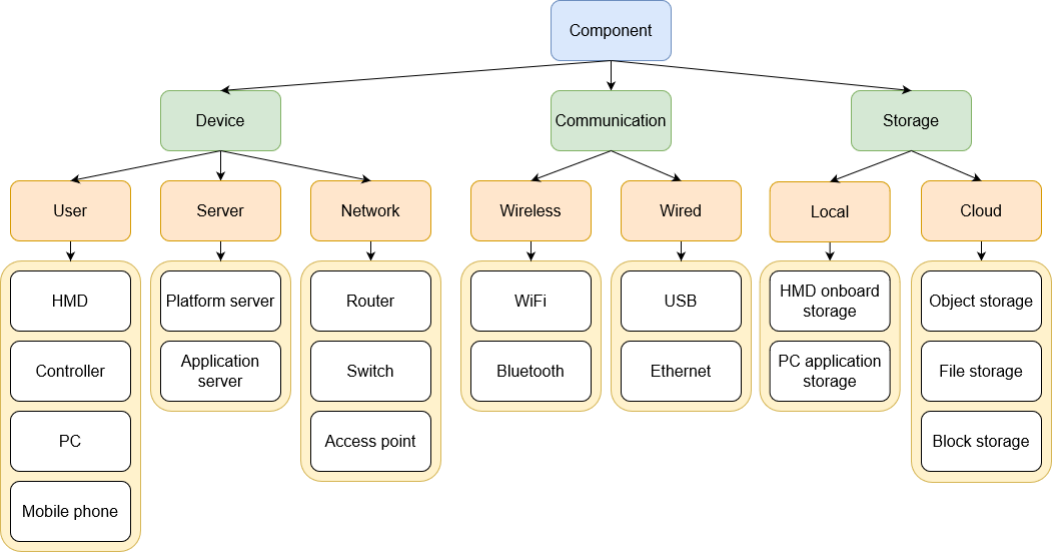
Classification of extended reality component types. HMD: head-mounted display.

A classification of XR component types can be seen in Figure 2, where components are broadly classified as either device, communication, or storage. Common components can be seen in yellow and are not limited to the ones shown. The XR proof-of-concept devices for experimental studies that identified either a security or privacy threat or a mitigation strategy for those devices were counted. Many studies used more than one proof-of-concept device type. Of the devices, 28 were XR HMD devices or other head-mounted eyewear (28/31), with many using the HTC Vive (8/31). However, most papers used a variety of other devices that were only used within one study, and two studies used a mobile phone device, the only non-HMD device. A summary of devices used can be seen in Table 6. All components tested and investigated for threats were thus user device components.

**Table 6.** Proof-of-concept device usage count across studies.

Proof-of-concept device	Number of papers
Oculus Rift	3
Meta Quest	5
Meta Quest 2	2
HTC Vive	7
Samsung Gear	2
Mobile	2
Other	10

**Figure 3.**
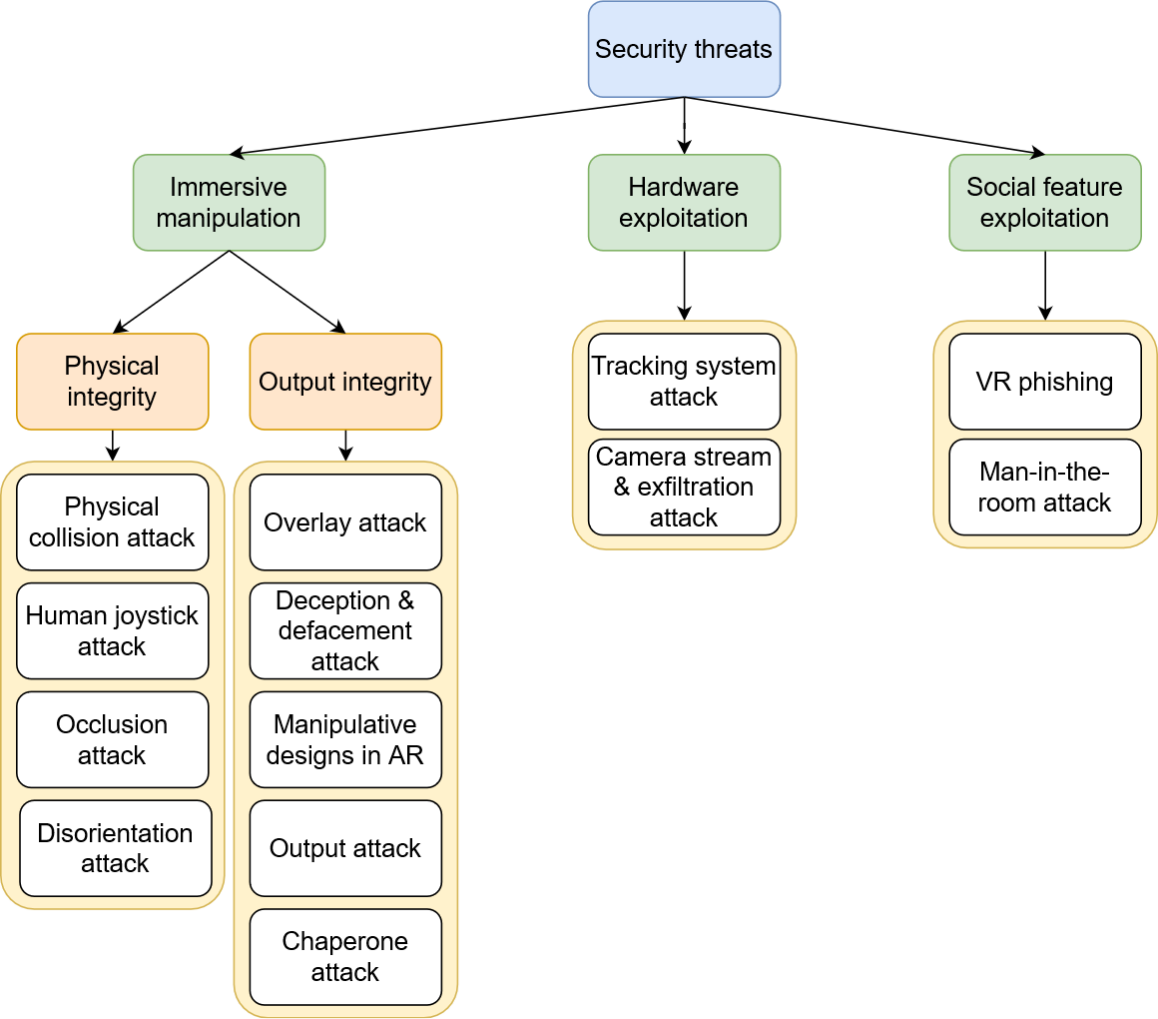
Security threat taxonomy and associated attacks. AR: augmented reality; VR: virtual reality.

We identified 20 unique attacks specific to an XR domain, as can be seen in the attack catalog in Multimedia Appendix 5. The security threat taxonomy and attacks classified in Figure 3 were developed from the literature review and by analyzing these attacks as described in Methods. Security threats specific to XR components are those that involve an explicit tampering, intrusion, or breakage of the system. They can be broadly classified as immersive manipulation, hardware exploitation, or social feature exploitation. Hardware exploitation encompasses any threat or attack that targets the unique hardware features of an XR system to degrade or intrude. Social feature exploitation includes any threat or attack that targets the unique social features of an XR system.

Immersive manipulation constitutes the majority of threats and cyberattacks identified in the literature. This encompasses any threat that utilizes the unique features of an XR session to target the security of the system or safety of the user. Threats that target physical integrity are those that aim to physically manipulate or impact a user, such as by causing cybersickness and physical collision with real-world objects [33,34]. Threats that target output integrity include any threats aiming to impact or degrade the sensory features of an immersive session. They include deception and defacement attacks, which were found to have the potential to impact users’ mental and emotional well-being by outputting inappropriate or upsetting visual or audio content [33,35].

**Figure 4.**
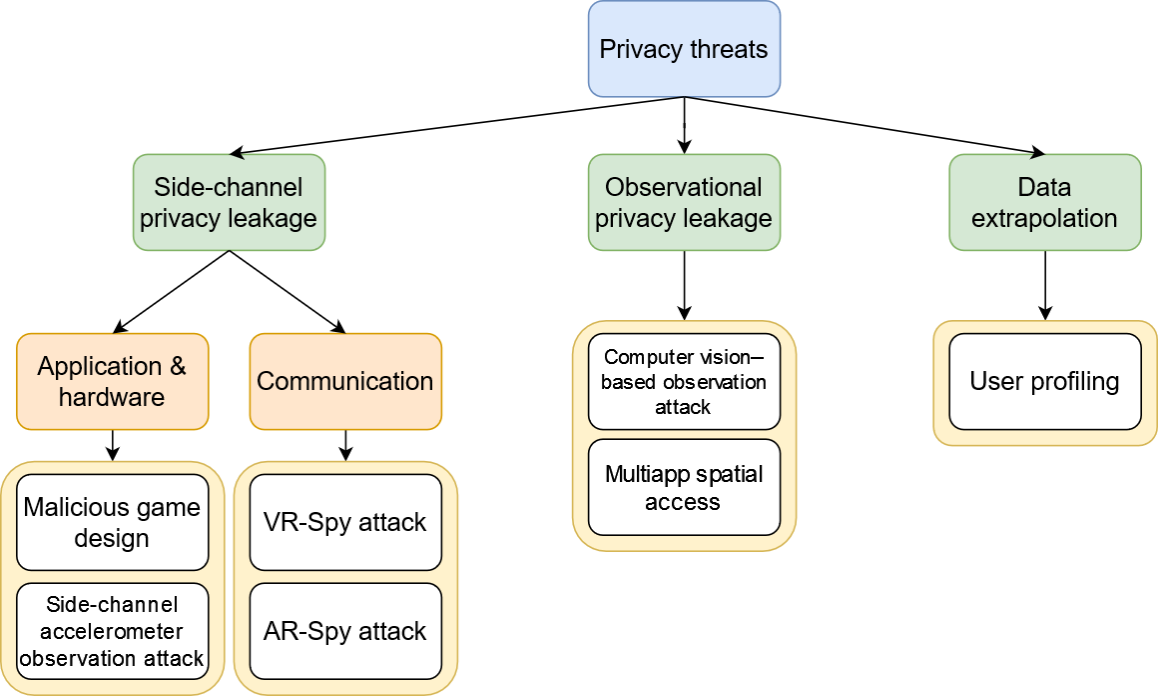
Privacy threat taxonomy and associated attacks.

The privacy threat taxonomy and associated attacks seen in Figure 4 were developed from the literature review as described in Methods. Privacy threats to XR components are those that are intended to passively disclose information with limited to no tampering or breaking of the system. They can be broadly classified as side-channel privacy leakage, observational privacy leakage, or data extrapolation. Privacy leakage refers to any confidential information that is inadvertently leaked and not obtained through an intrusion or premeditated attack on a system. Side-channel privacy leakage refers to privacy leakage that happens as an unintended result of the technical design of the system. This privacy leakage can either happen at the application and hardware level or communication level. Observational privacy leakage encompasses attacks that use physical observation of an XR user, or an XR user observing bystanders and the environment. Data extrapolation includes attacks that infer additional attributes and characteristics of a user, their environment, or a system from collected XR data.

The type of information that can be collected and inferred about users in these attacks includes biometric data, psychological data, and personally identifiable information such as [35,36] color blindness, mental and physical disability status, physical fitness, mental acuity, eyesight, and visual acuity. The study by Nair et al [36] demonstrated a side-channel privacy leakage attack with a VR game that can inconspicuously harvest such health-related data. A user profiling framework involving data collection and AI models to extrapolate such data was developed by Tricomi et al [37], with high mental workload tasks with eye tracking found to be the most effective combination for data collection. These attacks generally do not require any existing information related to the user to be stored on the device or for there to be a link to their social media account. These privacy attacks only require the data generated by the XR system or observation of the environment or user while they are using the system.

Cyber threats to XR components or the users of them were also classified in terms of the STRIDE model. A cyberattack can be classified into more than one category in the model. Of these cyberattacks, the most common threat was information disclosure, representing 50% (10/20) of attacks. The second biggest threat was tampering (45%, 9/20) and the smallest threat was repudiation (no attacks).

**Figure 5.**
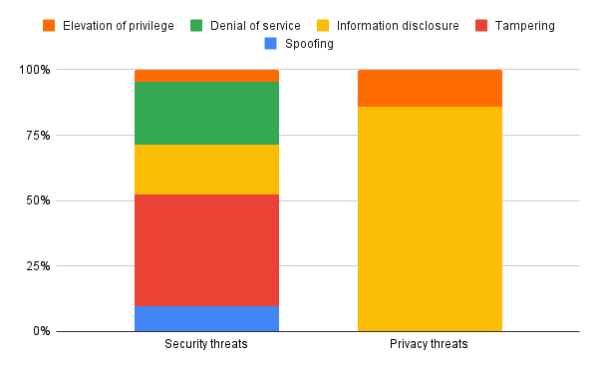
Constitution of security and privacy threats by STRIDE classification.

The constitution of the total number of security and privacy attacks by their STRIDE count can be seen in Figure 5. The majority of security threats are also tampering threats, and the majority of privacy threats are also information disclosure threats.

Garrido et al [38] and Warin and Reinhardt [39] developed an attacker privilege level classification for VR-specific attacks. A summary of the observable data attributes and data sources for each level can be seen in Multimedia Appendix 6. There are 4 different types of attackers: a hardware-level attacker (privilege attacker I) can access the low-level input and output signals of the HMD device; a client-level attacker (privilege attacker II) operates at an application level and can access the system abstract programming interfaces; a server-level attacker (privilege attacker III) can access or control the application servers; and a user-level attacker (non-privilege attacker) includes any attacker that can access public telemetry of the user in a social application. Of the 20 attacks, the most common level they occur at is the client level (70% of attacks, 14/20), and the least common attacker level is server (10% of attacks, 2/20). Some studies explicitly stated the privilege level of their identified attacks, and those that did not were categorized based on their features.

Only 3 studies described cybersecurity and privacy issues of XR in a health care context [32,40,41]. Letafati and Otoum [32] explored the security and privacy risks and potential mitigations of incorporating health care capabilities into the Metaverse as it relates to patient data flow and processing, distributed health AI/ML, and patient interaction. They identified the primary challenges with these technologies being maintaining the security of data as it is collected and processed, the privacy of these datasets, and the privacy of users as they are monitored in virtual therapy [32]. The NIST Privacy Framework is a tool for managing risk to an organization and improving governance of data it stores and processes. There are 4 tiers of framework implementation that delineate to what extent controls are in place to mitigate privacy risks, the lowest tier being that an organization is risk-informed but implements no controls to safeguard privacy. Health care organizations often exist at the second tier due to valuing data availability over confidentiality, which means limited mitigations are implemented and user AR data are at a higher risk than necessary [41]. Ara et al [40] also identified data security as being one of the greatest challenges for using AR in health care services.

Cybersecurity and privacy threats to VR systems identified in the threat taxonomies and their potential risk in a health care setting can be seen in Table 7, which maps these threats against an ERM model for managing organizational risk. The likelihood and impact of each risk will be highly dependent on the specific health care context and conditions of use of the VR system.

**Table 7.** Enterprise risk management framework mapping of extended reality cybersecurity risks for health care.

Category	Scope	Risk
Compliance	Risk to the organization’s ability to meet legal and regulatory obligations	An immersive manipulation attack that implicates personal safety or well-being can have legal consequences for the organization Privacy leakage and data extrapolation attacks can potentially expose or deanonymize user health data stored or collected by the organization Observational privacy leakage attack can implicate user, clinician, and organization privacy Lack of awareness of cybersecurity and privacy risks can reduce compliance to cybersecurity policies, which can impact organizational ability to meet legal and regulatory requirements
Service delivery and capacity	Risk to organization operations and ability to deliver services	An immersive cyberattack that impacts personal safety or well-being can also impact recovery and rehabilitation Immersive manipulation or hardware exploitation can render the VR system unusable, requiring a disruptive transition to alternate forms of intervention and treatment, which may not be as effective The impact of a cyberattack (eg, denial-of-service attack) on a virtual reality system can potentially spread and take down other organization resources, services, and access to health data
Community and stakeholder confidence	Risk to community and stakeholder confidence and ability to plan and create policies in relation to them	Cyberattacks can reduce community and stakeholder confidence in organizational ability to protect patients
Financial	Risk to financial performance, and how variance to this can impact the organization	Cyberattacks that implicate user privacy, safety, or treatment outcomes (eg, immersive manipulation attacks) can cause significant financial consequences for an organization, for example by reducing hospital funding or through legal events
Workplace health and safety	Risk to patients, staff, and the organization from workplace health and safety events	An immersive manipulation cyberattack can negatively impact user and staff safety and well-being, both physical and mental

In summary, the most common cyber threats to the XR environment reported in the reviewed literature according to the STRIDE model are information disclosure (10/20) followed by tampering (9/20), with most attacks taking place at the client level. Most privacy threats as defined by the developed taxonomies are also information disclosure threats, where user biometric, psychological, and identifying information can be collected through privacy leakage and lead to individual profiling. These threats can impact a health care organization financially and in terms of compliance and community and stakeholder confidence. Most security threats as defined by the taxonomies are tampering threats, the majority of which pose risks to device output integrity and user safety and well-being through immersive manipulation attacks. These threats can impact a health care organization in terms of compliance, service delivery and capacity, community and stakeholder confidence, finances, and workplace health and safety.

### RQ2: How Can Cybersecurity Risks to Clinical XR Systems, In Particular VR Systems, Be Reduced?

Cybersecurity and privacy risks to XR systems were identified in RQ1. RQ2 was answered by mapping the risk mitigation technologies and frameworks against the STRIDE framework to determine which mitigations may mitigate the cyber threats and attacks identified in RQ1, as seen in Table 8. These mitigations were also mapped against the NIST Cybersecurity Framework to identify how and where these mitigations may improve the security posture of a health care organization, as seen in Table 9. NIST is comprised of 5 core functions for organizations to organize their cybersecurity measures: identify, protect, detect, respond, and recover.

**Table 8.** Mitigating technologies mapped to STRIDE threat type.

STRIDE threat	Mitigation	Mitigating technology
Spoofing	Authenticity	Biometric and continuous authentication [38,44]
Tampering	Integrity	Trusted execution environments [36] Virtual reality vulnerability detection and prevention [42] Adversarial machine learning models to defend against data poisoning [32,36] Application integrity checks [33] Static and dynamic analyzers [38] Intrusion detection systems [45-47,59] Health care intelligent security model [40]
Repudiation	Nonrepudiability	Hardware usage indicators [48]
Information disclosure	Confidentiality	Differential privacy [32,36] Behavioral modifications [36] Location-based extended reality usage restrictions to dynamically defend against observational privacy leakage [43] Automated contextual awareness and response [44] Health care intelligent security model [40]
Denial of service	Availability	Design with the principle of redundancy [34] Intrusion detection systems [45-47,59] Health care intelligent security model [40]
Elevation of privilege	Authorization	Application access control [31,33,43,47-50] Design with the principle of least privilege [34,51]

**Table 9.** NIST^a^ cybersecurity framework mapping of mitigations for a health care organization.

Function	Scope	Mitigating technology or framework
Identify	Identify all components in the clinical system. Understand the roles and access levels of everyone that interacts with the system. Identify threats, assets, and risk. Create incident response plans.	ISO 27001/27002/27701 Framework [41] NIST Privacy Framework [41] Legislation and regulations [37,41] Collaboration with users, experts, and developers [37,52] Privacy policies [35,52] Threat modeling and risk assessment [34,47,53,54] Health care data modeling [40] Penetration testing [42]
Protect	Implement defensive technologies that protect system components. Create cybersecurity training and awareness among those that interact with the system. Manage access control and secure data processes.	Differential privacy [32,36] Behavioral modifications [36] Trusted execution environments [36] Application access control [31,33,43,48-50] Biometric and continuous authentication [38,44] Virtual reality vulnerability detection and prevention [42] Design with the principle of least privilege [34,51] Education and training [31] Design with the principle of redundancy [34]
Detect	Monitor system for unauthorized access and cybersecurity attacks to components. Investigate unusual activity.	Intrusion detection systems [45-47,59] Static and dynamic analyzers [38] Application integrity checks [33] Hardware usage indicators [48] Health care intelligent security model [40]
Respond	Prepare for responding to a cyber incident and minimizing its effect. Respond to cyber incidents through mitigating technologies. Manage communication routes. Plan for notifying stakeholders. Report incident to relevant authorities.	Adversarial machine learning models to defend against data poisoning [32,36] Location-based extended reality usage restrictions to dynamically defend against observational privacy leakage [43] Automated contextual awareness and response [44] Meta crime investigation [35] Health care intelligent security model [40]
Recover	Repair and restore extended reality components and system after a cyber incident. Plan for effective public and stakeholder assurance. Implement lessons learned into framework. Improve organizational resilience.	Code of ethics based on stakeholder perceptions [52]

a NIST: National Institute of Standards and Technology.

We found that 18/29 studies (62%) discussed a cyber defence technology or mitigation strategy. These were mapped to a STRIDE category based on the attribute provided by their functionality: authenticity, integrity, nonrepudiability, confidentiality, availability, or authorization. According to the STRIDE framework, threats of a specific STRIDE type may be mitigated by one of the corresponding technologies. For example, designing applications with the principle of least privilege can mitigate elevation of privilege threats such as man-in-the-room attacks [42], to restrict the ability of the attacker to gain access to private VR rooms. Intrusion detection systems can detect anomalous system resource usage indicative of tampering threats like a disorientation attack [33]. Location-based XR usage restrictions can respond to information disclosure threats, such as by restricting mobile AR usage in a hospital to protect occupant privacy [43].

Cyber defense technologies and mitigation strategies can also be mapped against 3 of these functions: protect, detect, and respond. Papers could identify mitigations of more than one function thus some papers were counted more than once. Some studies explicitly stated the function of their identified technology or strategy, and those that did not were classified based on their stated features. The majority (18 papers) identified a protection function. The next most common defensive technology or strategy was detection (9 papers), followed by response (5 papers). Implementing these technologies and strategies may mitigate risk to a health care XR system but none of these mitigations have been tested in a clinical context.

In addition to the defensive technologies, management- and operational-level regulations and frameworks will help to identify and mitigate the risks associated with VR/XR systems. One study by King et al [41] identified existing privacy laws, regulations, and frameworks to protect user data privacy in AR. Challenges in these privacy mechanisms include poorly defined data types, inadequate restrictions defined on these data types, improperly defined regulations, and information that should not be collected being undefined. Privacy frameworks to protect consumer information include the ISO 27001/27002/27701 Framework and NIST Privacy Framework. The purpose of the former is to protect personally identifiable information rather than specific information related to health care, while the latter is designed to improve the security and privacy posture of an organization. There are 4 tiers of framework implementation, which delineate to what extent controls are in place to mitigate privacy risks, where the higher the tier, the more controls and protections are in place. Health care organizations often exist at the second tier; by implementing the framework to a higher tier, organizations may be able to enhance data security [41]. These measures fulfill the NIST Framework identify function.

Adams et al [52] developed a VR code of ethics based on the perceptions of users and developers and in collaboration with them, the only instance of the recover function. Only two papers described defensive technologies explicitly applicable to health care services [32,40]. Ara et al [40] proposed a concept for an intelligent security model for a health care organization for detection of and protection against AR-based cybersecurity threats. Physical layer security, secure semantic communication, differential privacy, adversarial machine learning, and privacy bubbles are recommended tools to protect Metaverse-enabled health services [32].

## Discussion

### Principal Findings

The results of the literature survey indicate that the greatest threats to XR systems in terms of the STRIDE model are information disclosure followed by tampering. These constitute the majority of privacy threats (information disclosure) and security threats (tampering), as defined by the threat taxonomies. Relevant risks of these if VR is used in health care services are confidentiality being breached and user safety and well-being being put at risk. These risks can impact a health care organization in terms of compliance, service delivery and capacity, community and stakeholder confidence, and finances. Identified mitigations were classified using the STRIDE framework, the majority of which provided integrity followed by confidentiality, and potentially addressed the two most common STRIDE threats identified. When mapped against the NIST Cybersecurity Framework, most of these mitigations would fulfill a protective function.

The majority of attacks are an information disclosure threat, and they constitute the majority of privacy threats. These threats are certainly not unique to health care, having been described in the literature for a variety of XR applications such as in [2,3] and [13], but the consequences for health care can be unique: exposure of patient or staff data; impact on safety and well-being of persons; and legal, financial, and reputational damage. Additionally, it has been shown that VR systems collect a large amount of data from which biometric, psychological, and health-related information can be inferred by an attacker, such as color blindness, mental and physical disability status, physical fitness, and mental acuity [35,36]. This can potentially have severe and unique consequences for a health care VR system. For example, a user undergoing physical rehabilitation can possibly have some data exposed. By the nature of the treatment, they will likely be conducting a range of physical movements that are conducive to inferring certain data types identified by Nair et al [36], such as standing poses to measure anthroprometrics and reaction time by measuring the interval between a stimulus and motor response. A malicious actor could tamper with the application to monitor and exfiltrate the data generated during these sessions in a side-channel privacy leakage attack.

Tampering threats are the next most prolific STRIDE threat identified and they constitute the majority of security threats. Guzman et al [3], in their literature review of security and privacy approaches in MR, flag that current systems have poor output access control, contributing to both visual tampering (such as altering renders) and audio tampering (such as altering audio cues or instructions). Most security cyberattacks can be categorized as immersive manipulation, and in a health care setting, these attacks can negatively impact a user’s safety, such as by outputting distressing content or causing a user to physically collide with another object by similar tampering. For example, a user in occupational therapy may be exposed to an immersive manipulation physical integrity attack that causes them to collide with an object or health staff in the vicinity. All these cases could negatively impact not only user treatment outcomes, but also their physical and mental health by exacerbating or triggering existing symptoms or creating new health issues altogether. Cybersecurity is a dimension that should be incorporated in risk assessments when considering implementing XR in hospitals, with considerations on the compliance with applicable regulatory measures; the ability and capacity to deliver health services; the confidence of the community and stakeholders in the health care organization; financial performance; and workplace health and safety.

Most of the XR and VR components that were studied for security and privacy issues were HMDs, and the majority of these were the HTC Vive. Thus, current literature focuses heavily on a single type of end-user component and is limited in understanding of security and privacy issues with other components, such as threats to the XR server architecture. The potential risk to other components, including integration with other health care systems, should be investigated for a more holistic understanding of the threat landscape, as retrofitting cybersecurity and privacy controls to deployed systems is challenging [3].

Types of cybersecurity mitigation tools include protective, detective, and responsive strategies and technologies, such as access control and intrusion detection systems. Most of the mitigations identified in the review provide integrity, potentially reducing the risk of tampering threats. The next most common attribute provided by identified mitigations is confidentiality, which may be effective in mitigating information disclosure threats. These were the two highest risk and most common security and privacy STRIDE threats, respectively. Most of these technologies have not been developed beyond a proof-of-concept and none have been tested in a health care setting. As such, they likely require further investigation into their safety and reliability before they can be used and developed for health care applications. Additionally, the practical challenges and feasibility of implementing them must be understood. The modalities of implementing a mitigation strategy will be highly dependent on the specific health care context and conditions of use of the XR system.

For example, local differential privacy was one proposed mitigation identified in the scoping review that provides confidentiality [32,36]. It protects users from virtual monitoring and identification by adding a degree of error to a user’s virtual avatar, so it does not map directly to their physical features. The more error is added, the more likely a user is protected from monitoring, but the more usability is impacted as a user’s mental mapping of their physical movements to virtual is changed. This may have side effects or implications for an occupational therapy setting both in terms of effectiveness of treatment and accuracy of data collection and user progress tracking. The degree of error would need to be fine-tuned for the application or treatment, and the usability trade-off [36] may mean in some circumstances this mitigation may not be suitable at all.

The ethical considerations of using XR in health care are not the focus of this paper but must also be addressed before mainstream adoption of this technology into health care. XR has a range of beneficial usages in health care where it has been shown to improve patient outcomes, such as in occupational therapy where VR is effective to engage users. Like most major technology, it is likely that XR and VR will be integrated into the health care industry just as they are being integrated into many other major industries, regardless of their issues in terms of data security and privacy. Adams et al [52] propose a code of ethics for VR developers, encompassing security, privacy, and well-being dimensions and articulating ten principles that show alignment with requirements for quality, safety, and performance in hospitals: (1) do no harm, (2) secure the experience, (3) be transparent about data collection, (4) ask for permission, (5) keep the nausea away, (6) diversity of representation, (7) social spaces, (8) accessibility for all, (9) user centric design and experience, and (10) proactive innovation [52]. In their systematic review of cybersecurity threats of VR and AR, Alismail et al [24] conclude that effective mitigation techniques for VR and AR threats are adopting such a “code of ethics,” which is outside of the control of hospitals and users, and these techniques also include adopting a risk assessment approach, as we have articulated above [24].

Another proposed mitigation was biometric-based authentication [38,44], which leverages the data collection capabilities of HMDs to authenticate users based on biometric attributes like movement, providing authenticity, and mitigating some elevation of privilege threats according to the STRIDE framework. This replaces traditional authentication methods like passwords, which create overhead on the user and rely on them to create and manage the security of passwords. This is inherently insecure. Biometric-based authentication removes this psychological overhead on the user to manage their own security, but for users whose physical mobility may differ from the norm, the effectiveness of this technology may not be the same. Such a mitigation may pose ethical challenges as well as implementation challenges due to potential risk from the exposure of this biometric data. Dick [60] flags that in addition to transparency and disclosure practices, user privacy controls, and information security standards, guidelines and risk assessments should consider the unique risks presented by biometric identifying information.

This risk to data security has been acknowledged and discussed in the literature but the risk to user safety has not. These risks are heightened in a health care setting where attributes can more easily be inferred from VR usage data, and users are potentially more vulnerable. The current health-related data types that can be inferred from VR-generated sensor data like visual acuity, mental capability, and degree of physical fitness are quite general, but cyberattacks may evolve in the future to target more specific health-related attributes and threaten user confidentiality, especially as VR technology is further adopted into health care and gains more public awareness. Immersive manipulation cyberattacks have a similar potential to become more sophisticated and effective in achieving a range of malicious goals, such as undermining treatment outcomes by tampering with clinical VR devices.

These attacks do not rely on any preexisting personal or hospital details stored on the system or even a history of VR usage on the device. These attacks include novel methods unique to XR to gain access to confidential personal, physiological, and health-related attributes about an individual. Regulations, legislations, and policies exist to protect users and professionals from traditional and general risks to privacy and safety—such as the Australian Health Practitioners Regulatory Agency Code of Conduct, which mandates patient safety be made a priority and risk management practices be followed [61], and Health Insurance Portability and Accountability Act legislation in the United States [19]—but these frameworks may not be designed for the novel, unique, and growing threats posed by XR. This may pose legal and ethical dangers for a health care organization, as also mentioned by Morimoto et al [21]. This evolving threat landscape may make the ethical considerations of using XR technology in health care more complex and highlights the fact that the cybersecurity posture in all domains of health care must be proactive rather than reactive to avoid legal, ethical, privacy, and safety problems before they occur.

There has been more primary work done on the security and privacy issues of XR technology in recent years, peaking from 2020 to 2023 as seen in Multimedia Appendix 3. There have also been many literature reviews and secondary analyses similar to this work that were not included in this scoping review. Unlike these existing secondary analyses and as far as the authors are aware, there is little literature focusing on the specific cybersecurity challenges and risks of using XR technology in a health care context or potential countermeasures against such risks. As such, this scoping review contributed the following: (1) taxonomic analysis and classification of threats and cyberattacks that may be applicable to health care XR applications, (2) STRIDE analysis and classification of threats and countermeasures that may be applicable to health care XR applications, (3) identification of potentially high risk threats to health care XR technologies, (4) ERM mapping of cybersecurity risks and challenges for a health care organization implementing XR, and (5) NIST Cybersecurity Framework mapping of risk mitigations for a health care organization implementing XR.

### Limitations

A potential limitation of this scoping review was the variety and quality of results and scholarly sources Google Scholar returned. Any high-quality or important sources in the field not indexed by the search engine were omitted. This aspect of the study could possibly be strengthened in future work by searching from a range of other scholarly sources.

### Conclusion

The most significant threats posited for a health care VR system in terms of the STRIDE framework were information disclosure followed by tampering threats. VR systems generate a large amount of data from the sensors on board, data which may be used to infer additional attributes about users including personal and health-related attributes, such as physical fitness. This poses real risks in clinical environments, as privacy attacks may lead to breaches of user confidentiality. Immersive manipulation attacks constitute the majority of security threats. If a clinical VR device is compromised by a cyberattacker, they can tamper with the delivery and content of a clinical VR immersive session, which may affect the delivery of care and potentially the safety and well-being of individuals. These risks have implications on regulatory compliance, health service delivery, communities, staff health and safety, and the financial performance of a health care organization. The majority of mitigations identified for these threats address information disclosure and tampering and provide protective capabilities for an organization according to the NIST Cybersecurity Framework. However, only 3 of 29 included papers mentioned health care and none of the threats and mitigations identified have been studied in or assessed for health care. This can present ethical and practical implementation challenges for a health care organization. The specific cybersecurity and privacy risks presented by XR technology should be considered as a part of system-wide digital risk management frameworks by health organizations, within their proposed context of use, intended purpose, and perceived benefits to health care delivery and individuals.

### Future Directions

The specific cybersecurity and privacy risks related to XR technology used in health care services require dedicated studies. Due to the wide range of clinical applications of this technology, the risks for each health service should be studied individually. Only threats to end-user devices were investigated. Considering XR systems usually involve more than one component, future work can also study the threats to other XR components such as the controllers and servers. Mitigation strategies and technologies currently suggested by the literature must be assessed for their practical feasibility and effectiveness for health care applications. The effectiveness of existing regulations, frameworks, and policies in health care for assessing and mitigating the unique risks posed by XR technology to user privacy and well-being also requires investigation.

## Supplementary material

10.2196/59409 Multimedia Appendix 1 Electronic database search strategy.

10.2196/59409 Multimedia Appendix 2 Summary of papers.

10.2196/59409 Multimedia Appendix 3 Studies published per year.

10.2196/59409 Multimedia Appendix 4 Glossary.

10.2196/59409 Multimedia Appendix 5 Cyber attack catalogue.

10.2196/59409 Multimedia Appendix 6 Privilege level summary.

10.2196/59409 Checklist 1 PRISMA-ScR (Preferred Reporting Items for Systematic reviews and Meta-Analyses extension for Scoping Reviews) checklist.

## Abbreviations

AI: artificial intelligence
AR: augmented reality
DoS: denial of service
ERM: enterprise risk management
HMD: head-mounted display
ML: machine learning
MR: mixed reality
NIST: National Institute of Standards and Technology
PRISMA-ScR: Preferred Reporting Items for Systematic reviews and Meta-Analyses extension for Scoping Reviews
STRIDE: spoofing, tampering, repudiation, information disclosure, denial of service, and elevation of privilege
VR: virtual reality
XR: extended reality
