# Supplementary material for: Cybersecurity and Privacy Issues in Extended Reality Health Care Applications: Scoping Review
Source: JMIR XR Spat Comput. 2024 Oct 17;1:e59409. doi: 10.2196/59409 (PMC13202513; doi:10.2196/59409)
Supplement: Multimedia Appendix 1 [file xr-v1-e59409-s001.doc]

# Appendix 1 – Search strategy

## Aim

The goal of the search strategy was to identify the most important literature on cybersecurity challenges and countermeasures for general-use and healthcare XR technology, with an emphasis on VR.

## Database

There are many scholarly databases that specialise on topics relevant to the scope of this work. These include and are not limited to healthcare sciences in PubMed and social science in JSTOR. Google Scholar is a database that indexes literature across disciplines and topics, and so was chosen as the source for this work to find a balance of literature and increase the likelihood of not missing important intersectional work. Additionally, due to the enormous number of initial results returned by Google Scholar alone, another database search was not deemed necessary.

## Search strategy

Search terms were defined with guidance from the cybersecurity expert on the team. The original search terms were drawn directly from the topic and each needed to occur in conjunction with at least one of the following keywords: virtual reality, augmented reality, mixed reality or extended reality. They could occur anywhere in the text for the paper to be returned:

- security OR privacy
- healthcare AND (security OR privacy)

These returned 69 200 results, too many results that one author could feasibly screen, so they were refined to return more precise results. When the search was limited for these same key words only in the title, it returned 387 papers, with only 1 returned with the healthcare search. This was considered too few papers, with not enough content related to healthcare. The search terms were then broadened to include more cybersecurity topics, as well as healthcare and other non-standard architectures. Like the original search terms, these had to occur with at least one of the following keywords in the title: virtual reality, augmented reality, mixed reality or extended reality.

Cybersecurity-centric search term words:

- privacy OR security OR attack OR threat OR secure OR securing

Specific cybersecurity issues identified to be of relevance or special interest to the topic:

- access control OR side-channel OR user profiling OR tracking user location OR dark designs

Search terms for analyses on XR issues specific to healthcare and non-standard architectures:

- survey architectures OR analysis healthcare

These refined search terms returned 482 papers, which included multiple related to healthcare. They were manually screened by one author (KL) based on title and abstract. If relevance was unclear from the title of the paper only, abstract would then be evaluated to determine eligibility. References of these studies were also checked to identify any other significant and relevant literature not included in the results, in which case not all keywords needed to appear in the title. 53 studies passed screening and the Covidence research review management software was used to facilitate full-text review. This was conducted independently by three authors (KL, MD and AMK) against the inclusion and exclusion criteria. Conflicts were resolved by all authors through discussion and then agreement.

## Eligibility criteria

The criteria for a study to be included are as follows.

- The publication year is between 2017 and 2024.
- The issue or mitigation described in the study is relevant and applicable to XR and is well described.
- The terms of the search request appear in the title of the study as specified in the search strategy (see Search strategy section).

The criteria for a study to be excluded are as follows. Only one criterion must be met for a study to be excluded:.

- The publication date is before 2017.
- The study is a literature survey, viewpoint piece or opinion piece.
- The issue or mitigation described in the study is not relevant or applicable to XR or is poorly described.
- The study is not peer-reviewed.
- The study is on VR but is not relevant to the HMD VR platform.

## Data charting and extraction

The following data was extracted from the included studies:

## Publication information

## Authors

## Year of publication

## Topical information

## XR domains identified in the title, or ‘Other’ if none

## Major contribution made by the study, identified by the author as being at least one of the following:

- - - Novel security threat
    - Novel privacy threat
    - Mitigation identified
    - Taxonomic analysis
    - User experience evaluation
    - Other

## Cyber threat information

## For each cyber attack or threat identified, the following was extracted:

- - - Name
    - Description
    - If a proof-of-concept was provided for the attack, what physical devices were used
    - Whether each cyber attack is an active (security) threat or a passive (privacy) threat
    - What XR features the attack exploits
    - The intended effect of the attack
    - In what XR domains the attack is described as effective e.g. AR, VR, MR or XR

## Privilege level of the attack, based on attack description if unspecified

## STRIDE classifications, based on attack description if unspecified

## Risk mitigation information:

## For each defence technology or strategy identified, the following was extracted:

- - - Name
    - Description
    - NIST Cybersecurity classifications, based on its description if not explicitly stated
    - STRIDE classifications, based on its description if not explicitly stated
    - Whether it has been tested or simulated in a healthcare setting

## Search strategy overview

The search strategy is summarised in Table 1.

Table 1. Study collection and exclusion process

| Collected | |
| --- | --- |
| Search terms | No. |
| (privacy OR security OR attack OR threat OR secure OR securing) AND (Virtual reality OR augmented reality OR mixed reality OR extended reality) | 451 |
| (access control OR side-channel OR user profiling OR tracking user location OR dark designs) AND (Virtual reality OR augmented reality OR mixed reality OR extended reality) | 13 |
| (survey architectures OR analysis healthcare) AND (Virtual reality OR augmented reality OR mixed reality OR extended reality) | 18 |
| Excluded | |
| Reason | No. |
| Abstract Screening | 425 |
| Paper unreliable or low quality | 13 |
| Viewpoint/Opinion piece | 5 |
| Literature survey/review | 2 |
| Outside of project scope | 2 |
| Published before 2017 | 2 |
