# Supplementary material for: Cybersecurity and Privacy Issues in Extended Reality Health Care Applications: Scoping Review
Source: JMIR XR Spat Comput. 2024 Oct 17;1:e59409. doi: 10.2196/59409 (PMC13202513; doi:10.2196/59409)
Supplement: Multimedia Appendix 2 [file xr-v1-e59409-s002.doc]

| **Title** | **Summary** | **XR Dimension** | **Contribution** | **Reference** |
| --- | --- | --- | --- | --- |
| Security, Privacy and Safety Risk Assessment for Virtual Reality Learning Environment Applications | Constructs a threat model and risk assessment for threats to a social VLRE server (vSocial). It was found packet sniffing, packet redirection and lack of user notification had the greatest impact on user privacy. | VR | Novel security threat Novel privacy threat | Gulhane et al. 2019 [53] |
| Tracking attacks on virtual reality systems | An attack proof-of-concept was demonstrated on an active ground station, passive HMD in which a IR LED device interfered with the IR light positional system and jammed the tracking system. Two more proof-of-concepts were conducted that achieved the same outcome, with the last shown possible to also manipulate position & orientation. Countermeasures include intrusion detection and using an encrypted set of optical signals. | VR | Novel security threat | Rafique & Cheung 2020 [45] |
| Detection of Security and Privacy Attacks Disrupting User Immersive Experience in Virtual Reality Learning Environments | Investigates ways to detect and analyse SP (security & privacy) attacks at a network and application level using ML and statistical analysis that disrupt UIX (usability & immersive experience). SP attacks were found to cause a 56% decrease in immersion & 43% decrease in usability. | VR | Mitigation identified User experience evaluation | Valluripally et al. 2023 [47] |
| Attack trees for security and privacy in social virtual reality learning environments | Hardening and principle of least privilege was found to be the best design principle combination for increasing security, and diversity and principle of least privilege was the best combination for increasing privacy. Unauthorized access and causes of DoS attacks were the most vulnerable in regards to security, and user movement tracking and user location tracking were the most vulnerable in terms of privacy. | VR | Mitigation identified | Valluripally et al. 2020 [54] |
| A Survey on Mobile Augmented Reality With 5G Mobile Edge Computing: Architectures, Applications, and Technical Aspects | Localised MAR (mobile augmented reality) is much more secure and private than cloud-based because data it is not transferred outside the network. Security in MAR is classified into input, data access and output security. Data protection privacy is split into aggregation, processing, and storage, with linkability, detectability and identifiability as threats. | AR | Novel security threat Novel privacy threat Mitigation identified | Siriwardhana et al. 2021 [57] |
| Modeling and Defense of Social Virtual Reality Attacks Inducing Cybersickness | Security concerns in VRLE concern DDoS and unauthorised access attempt attacks. Privacy concerns regard man-in-the-room and packet tampering attacks to disclose confidential information. Safety concerns include XSS browser attacks, immersion attacks, and network fault attacks leading to cybersickness. | VR | Novel security threat Novel privacy threat Mitigation identified | Valluripally et al. 2022 [34] |
| Ethics Emerging: the Story of Privacy and Security Perceptions in Virtual Reality | Users and developers express concern related to well-being, security, and privacy. Both the HTC Vive and Oculus Rift have lacklustre privacy policies, with most applications entirely lacking them, and those that have them do not illustrate the type of VR data collected with any detail. | VR | User experience evaluation | Adams et al. 2018 [52] |
| A Case Study of Security and Privacy Threats from Augmented Reality (AR) | A simulated attack against alphanumeric passwords on a bystander's touch-device in which the attacker wore a Samsung Gear with an attached ZED camera whose video. Their password was able to be identified by modelling and processing of the video footage. | AR | Novel privacy threat | Chen et al. 2018 [56] |
| Securing Augmented Reality Output | An AR platform called Arya is designed, implemented and tested. It contains an 'output policy module' which sits between applications and output drivers, ensuring the security of outputs and safety of users, restricting what can be outputted to the AR environment. | AR | Mitigation identified | Lebeck et al. 2017 [49] |
| A systematic threat analysis and defense strategies for the metaverse and extended reality systems | Categorises and enumerates all potential security and privacy, safety and governance concerns in depth in XR. There are vulnerabilities not only in the XR components but XR development technologies. | AR, VR, XR, MR | Mitigation identified Taxonomic analysis | Qamar et al. 2023 [35] |
| SoK: Data Privacy in Virtual Reality | The risk of data attributes collected and stored in VR were measured using statistical methods, and it was found that room size, interpupillary distance (IPD) and height were at the greatest risk of data leakage and noise. Attacks on privacy are enumerated and defences recommended for each. | VR | Novel privacy threat Mitigation identified Taxonomic analysis | Garrido et al. 2024 [38] |
| Virtually secure: A taxonomic assessment of cybersecurity challenges in virtual reality environments | Classifies VR security challenges as Exploit, Breach, Impact and Intent. Cybersecurity defences can be categorised as: authentication, intrusion detection, cyber risk assessment and privacy preservation. | VR | Mitigation identified Taxonomic analysis | Odeleye et al. 2023 [46] |
| Threats faced by mixed reality & countermeasures | Mitigation strategies categorised as input protection, data protection, output protection, user interaction, and device protection. Threats identified for each category. | MR | Mitigation identified Taxonomic analysis | Syal & Mathew 2020 [58] |
| Discerning User Activity in Extended Reality Through Side-Channel Accelerometer Observations | Three different movements were discernible from the data produced by an accelerometer attached to a user's arm. It is suggested that this data could be used with other biometric and physiological data for a multi-modal biometric authentication. | XR | Novel privacy threat | Andrade et al. 2020 [44] |
| A Survey on Metaverse: Fundamentals, Security, and Privacy | On the Metaverse platform there are threats to authentication and access control, data management, and privacy, as well as network related threats. Threats are listed and described. | XR | Novel security threat Novel privacy threat Mitigation identified Taxonomic analysis | Wang et al. 2023 [59] |
| Vision: Usable Privacy for XR in the Era of the Metaverse | Threats to XR in the Metaverse are described and categorised into input threats, data threats, output threats, user-interaction threats, and device threats. A plan and central research questions for future research on usable XR privacy is outlined. | XR | Novel privacy threat Mitigation identified | Warin & Reinhardt 2022 [39] |
| PrivacyManager: An Access Control Framework for Mobile Augmented Reality Applications | PrivacyManager is a safety and privacy control framework for mobile AR applications. It allows entities other than the end user to protect their privacy and ensure the safety of the user and those around them, by implementing policies and rules of what an AR application is allowed to do in specific environments and situations. | AR | Mitigation identified | Lehman & Tan 2017 [43] |
| A Survey on Privacy Issues of Augmented Reality Applications | Privacy frameworks include ISO 27001/27002/27701 Framework and NIST Privacy Framework. Challenges in privacy mechanisms include poorly defined data types, inadequate restrictions defined on these data types, improperly defined regulations, and information that should not be collected being undefined. | AR | Novel privacy threat | King et al. 2020 [41] |
| ARSpy: Breaking Location-Based Multi-Player Augmented Reality Application for User Location Tracking | Network traffic in location-based AR applications can potentially leak private user location data. It was found that victim location and trajectory could be determined with an accuracy of at least 90% in both scenarios. Three strategies to mitigate the attack potential were recommended. | AR | Novel security threat Novel privacy threat Mitigation identified | Shang et al. 2022 [51] |
| Exploring the Privacy Risks of Adversarial VR Game Design | VR applications can be designed to harvest user information without consent, such as mental and physical characteristics. Such a game is designed and tested with VR users to provide a proof-of-concept of such an application, and the extent of the data that can be gathered. | VR | Novel security threat Novel privacy threat | Nair et al. 2023 [36] |
| VR-Spy: A Side-Channel Attack on Virtual Key-Logging in VR Headsets | VR-Spy uses channel state information (CSI) of Wi-Fi to monitor and recognise virtual key strokes with an average accuracy of 69.75%. The success of the attack depends on many factors. | VR | Novel security threat Novel privacy threat | Arafat et al. 2021 [55] |
| Privacy Leakage via Unrestricted Motion-Position Sensors in the Age of Virtual Reality: A Study of Snooping Typed Input on Virtual Keyboards | The sensor security provided by OpenVR SDK, Oculus Platform SDK, and WebXR Device API is investigated. Most of the sensor data does not require any user permissions. | VR | Novel security threat Novel privacy threat Mitigation identified | Wu et al. 2023 [48] |
| Security and Privacy Evaluation of Popular Augmented and Virtual Reality Technologies | The 10 most commonly available AR/VR devices and applications are investigated for privacy and security issues. Evaluation focused on device authentication, user profiling, access control and database security. | AR, VR | Novel security threat Novel privacy threat | Noah et al. 2022 [50] |
| Immersive virtual reality attacks and the human joystick | Chaperone attack modifies the VE boundaries; disorientation attack creates dizziness and confusion in user; human joystick attack controls users physical movements; overlay attack places unwanted content in the VE. Camera Stream and Tracking Exfiltration remotely enables and exfiltrates the camera stream. | VR | Novel security threat Novel privacy threat Mitigation identified | Casey et al. 2021 [33] |
| The Dark Side of Augmented Reality: Exploring Manipulative Designs in AR | Potential scenarios where user privacy, safety or security is compromised by dark patterns in AR are constructed and tested on users. Recommended strategies to mitigate the affect of dark patterns include raising awareness, educating users, and implementing an output control framework. | AR | Novel security threat | Wang et al. 2023 [31] |
| Rise of the Metaverse’s Immersive Virtual Reality Malware and the Man-in-the-Room Attack & Defenses | A Man-in-the-room attack is demonstrated on the Bigscreen Metaverse application. A tool suite to mitigate unsafe coding practices that cause the responsible vulnerabilities was developed as FOSS. | VR | Novel security threat Novel privacy threat Mitigation identified | Vondráček et al. 2023 [42] |
| You Can’t Hide Behind Your Headset: User Profiling in Augmented and Virtual Reality | A general framework for user identification and profiling in VR and AR using ML/AI technology is developed and compared across ML/AI algorithms. The framework involves four components: user data acquisition, bias removal, time series engineering and machine learning prediction. | AR, VR | Novel privacy threat | Tricomi et al. 2023 [37] |
| Comprehensive analysis of augmented reality technology in modern healthcare system | Data security was identified as the most significant challenge in regards to AR healthcare applications. An intelligent dynamic security model is proposed. | AR | Mitigation identified | Ara et al. 2021 [40] |
| Digital Healthcare in The Metaverse: Insights into Privacy and Security | Digital healthcare within the Metaverse potentially presents a variety of security and privacy challenges. These are ubiquitous data collection and communication, distributed medical AI and gamification and social activities in virtual therapies. Mitigation strategies identified for each. | XR | Novel privacy threat Mitigation identified | Letafati & Otoum 2023 [32] |
