# Supplementary figures and images for: Cybersecurity and Privacy Issues in Extended Reality Health Care Applications: Scoping Review
Source: JMIR XR Spat Comput. 2024 Oct 17;1:e59409. doi: 10.2196/59409 (PMC13202513; doi:10.2196/59409)

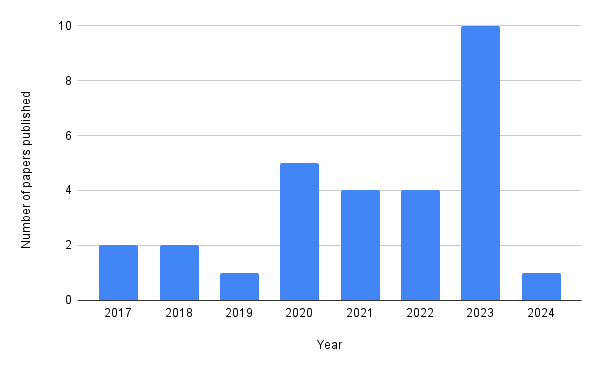

Supplement: Multimedia Appendix 3 [file xr-v1-e59409-s003.png]
