# Supplementary material for: Cybersecurity and Privacy Issues in Extended Reality Health Care Applications: Scoping Review
Source: JMIR XR Spat Comput. 2024 Oct 17;1:e59409. doi: 10.2196/59409 (PMC13202513; doi:10.2196/59409)
Supplement: Multimedia Appendix 4 [file xr-v1-e59409-s004.doc]

| **Term** | **Definition** | **Reference** |
| --- | --- | --- |
| Active attack | An attack that breaks or modifies an information system, or otherwise intrudes upon it without authorisation. |  |
| Application programming interface | A piece of code which allows one software component to use and interact with a different software component. |  |
| Augmented reality | A technology that augments the physical world by overlaying virtual features and functionality. |  |
| Cyber attack | A malicious pre-mediated intrusion or breaking of an information system using information technologies conducted by an individual or group. |  |
| Denial-of-service | Refers to the event in which an information system resource is made unavailable, generally as the result of a cyber attack. |  |
| ERM model | A framework for managing organisational risk. | [29] |
| Exploit | A program or other piece of technology that can target vulnerabilities in another system to launch a cyber attack. Alternatively, when vulnerabilities are utilised to break, intrude, or learn confidential information from a system. |  |
| Extended reality | A broad term used to refer to augmented reality, mixed reality and virtual reality, or a technology which combines the use of them. |  |
| Head-mounted display | A device worn over the head and eyes that generally provides visual and audio output to enable virtual reality. |  |
| Intrusion | When an attacker gains access or entry to an information system they are not authorised to. |  |
| Mixed reality | A technology in which an overlaid virtual world coexists and interacts with the physical one. |  |
| NIST Cybersecurity Framework | A framework comprised of five core functions for organisations to organise their cybersecurity measures: Identify, Protect, Detect, Respond, and Recover. | [30] |
| Passive attack | An attack that learns confidential information about a system without breaking or modifying it. |  |
| Privacy leakage | Confidential information that is invertedly leaked and not obtained with an active attack. |  |
| Proof-of-concept | A laboratory demonstration of a cyber attack or technology to verify its feasibility or potential. |  |
| STRIDE model | A common threat modelling framework developed by Microsoft with six categories of threats: spoofing, tampering, repudiation, information disclosure, denial of service and elevation of privilege. | [28] |
| Threat | The tangible risk that an information system will be compromised by an attacker. |  |
| Threat modelling | The process of identifying and evaluating threats to an information or physical system. |  |
| Virtual reality | A technology that generates a virtual world that a user can immerse themselves in. |  |
| Vulnerability | A security flaw in an information system an attacker can exploit. |  |
