# Supplementary material for: Cybersecurity and Privacy Issues in Extended Reality Health Care Applications: Scoping Review
Source: JMIR XR Spat Comput. 2024 Oct 17;1:e59409. doi: 10.2196/59409 (PMC13202513; doi:10.2196/59409)
Supplement: Multimedia Appendix 5 [file xr-v1-e59409-s005.doc]

| Name | STRIDE | Privilege level | XR Dimension | Proof-of-concept device | Description | Reference |
| --- | --- | --- | --- | --- | --- | --- |
| **Security** |  |  |  |  |  |  |
| Man-in-the-Room attack | I, E | II, III, IV | VR |  | An attacker exploits vulnerabilities in the VR/VE platform to gain unauthorised entry into a private virtual room where they can remain virtually invisible | [42] |
| Tracking system attack | T | IV | VR | HTC Vive | An attack in which an active ground station, passive HMD tracking system is jammed and manipulated | [45] |
| Occlusion attack | T, D | I, II | VR |  | An attack in which the user's VE view is obstructed or noise attenuation causes audio quality loss or discomfort. | [47] |
| Physical collision attack | T, D | I, II | VR |  | Any attack that causes the user to collide with a real world object or entity | [47] |
| Camera stream & exfiltration attack | I | I, II | VR | HTC Vive Oculus Rift | Any attack that intends to access and exfiltrate the stream from a front facing VR camera | [33] |
| Chaperone Attack | T | II | VR | HTC Vive Oculus Rift | Any attack that modifies the VE boundaries so that a user is unable to identify their real world boundaries | [33] |
| Output attack | T, D | I, II | VR |  | Any attack in which HMD output is manipulated without user consent | [35] |
| Deception & Defacement attack | T, D | I, II | VR |  | Any attack on HMD output such that it is manipulated to deceive or withhold information | [35] |
| Manipulative designs in AR | S | II | AR/VR |  | A purposeful design of the AR UI and UX to trick users into making decisions and behave in a manner they otherwise would not, usually with the intention of learning their private information | [31] |
| Human Joystick Attack | T | II | VR | HTC Vive Oculus Rift | Any attack that manipulates or controls a user's movement with the intent of sending them to a location they did not otherwise intend to go to | [33] |
| Disorientation attack | T | I, II | VR | HTC Vive Oculus Rift | Any attack with the intent of causing dizziness and confusion in the user | [33] |
| VR Phishing | S, I | IV | VR |  | A phishing attack using features unique to VR | [35] |
| Overlay attack | T, D | II | VR | HTC Vive Oculus Rift | Any attack that overlays unwanted content within the VE view that the user is unable to remove or move | [33] |
| **Privacy** |  |  |  |  |  |  |
| Malicious game design | I | II | VR | HTC Vive Vive Pro 2 Oculus Quest 2 | VR applications designed to maliciously harvest user information without consent | [36] |
| VR-Spy | I | IV | VR | Oculus Quest at 5G | VR-Spy uses channel state information (CSI) of WiFi to monitor and recognise virtual keystrokes within a VE with an average accuracy of 69.75% | [55] |
| Computer vision-based observation attack | I | IV | VR/AR | Samsung Gear ZED camera | An attack in which the attacker pretends to be immersed in a VR HMD and uses a camera added to the HMD or built-in to observe and derive private information about a bystander | [56] |
| Multiapp spatial access | I, E | II, IV | VR |  | An attack in which devices or avatars are discreetly placed to observe other avatars and VEs in a virtual space without the knowledge or permission of the occupants | [35] |
| User profiling | I | I | AR/VR | HTC VIVE Pro Eye Microsoft Hololens | An attack and general framework for user identification and profiling in VR and AR | [37] |
| Side-channel accelerometer observation attack | I | I | XR | Oculus Quest (software version 19) Android-based mobile device | An attack to identify the specific movements made by VR users from accelerometer data from an additional wearable device on their person during an immersive session using ML/AI techniques | [44] |
| AR-Spy | I | II, III | AR | Android mobile phone | AR-Spy is a side-channel attack framework and algorithm to determine user location and trajectory in applications where users can upload location-specific content | [51] |
