# Supplementary material for: Cybersecurity and Privacy Issues in Extended Reality Health Care Applications: Scoping Review
Source: JMIR XR Spat Comput. 2024 Oct 17;1:e59409. doi: 10.2196/59409 (PMC13202513; doi:10.2196/59409)
Supplement: Multimedia Appendix 6 [file xr-v1-e59409-s006.doc]

Multimedia Appendix 4. Privilege level summary.

|  | **Attacker type**^b^ | Privileged Attacker I | Privileged Attacker II | Privileged Attacker III | Non-Privileged Attacker |
| --- | --- | --- | --- | --- | --- |
|  | **Adversary**^c^ | Hardware (I) | Client (II) | Server (III) | User (IV) |
| Data sources | **Raw sensor data**^b^ | ✓ |  |  |  |
|  | **Processed Telemetry**^b^ | ✓ | ✓ |  |  |
|  | **Rendering pipeline & host system APIs**^b^ | ✓ | ✓ |  |  |
|  | **Networked Telemetry**^b^ |  | ✓ | ✓ |  |
|  | **Presented Telemetry**^b^ |  |  | ✓ | ✓ |
| Observable attribute classes | **Device**^b^ | ✓ | ✓ |  |  |
|  | **Network**^b,c^ |  | ✓ | ✓ |  |
|  | **Audio**^b,c^ | ✓ | ✓ | ✓ ^a^ | ✓ ^a^ |
|  | **Behaviour**^b,c^ | ✓ | ✓ | ✓ | ✓ |
|  | **Text**^c^ | ✓ | ✓ | ✓ | ✓ |
|  | **Video**^c^ | ✓ | ✓^a^ |  |  |
|  | **Physiological Signals**^c^ | ✓ | ✓ | ✓ |  |
|  | **System**^c^ | ✓ | ✓ | ✓ |  |
|  | **Geospatial & Inertial Telemetry**^b,c^ | ✓ | ✓^a^ | ✓^a^ | ✓^a^ |

^a^Observable only in lower quality/pre-processed/abstract format

^b^[39]

^c^[38]
